# A Ring-Type Triboelectric Nanogenerator for Rotational Mechanical Energy Harvesting and Self-Powered Rotational Speed Sensing

**DOI:** 10.3390/mi13040556

**Published:** 2022-03-31

**Authors:** Yida Xin, Taili Du, Changhong Liu, Zhiyuan Hu, Peiting Sun, Minyi Xu

**Affiliations:** 1 Dalian Key Lab of Marine Micro/Nano Energy and Self-Powered System, Marine Engineering College, Dalian Maritime University, Dalian 116026, China; xinyida@dlnu.edu.cn (Y.X.); dutaili@dlmu.edu.cn (T.D.); zhiyuanhu@dlmu.edu.cn (Z.H.); 2 College of Mechanical and Electronic Engineering, Dalian Minzu University, Dalian 116600, China; liuch@dlnu.edu.cn

**Keywords:** rotating machinery, triboelectric nanogenerator, rotational mechanical energy harvesting, self-powered sensor

## Abstract

In recent years, sensors have been moving towards the era of intelligence, miniaturization and low power consumption, but the power-supply problem has always been a key issue restricting the popularization and development of machine-mounted sensors on the rotating machinery. Herein, we develop a ring-type triboelectric nanogenerator (R-TENG) that functions as a sustainable power source as well as a self-powered rotational speed sensor for rotating machinery. The R-TENG adopts a freestanding mode and consists of a ring-type container unit, an end cover and polytetrafluoroethylene (PTFE) cylinders. In this study, the influence of the number of cylinders, the PTFE cylinder’s diameter and the rotational speed on the electrical output are systematically examined, and the motion law of the PTFE cylinders in the container is revealed by the experimental results and verified by kinetic simulation. At a rotational speed of 400 rpm, the output voltage, current and transferred charge of the designed R-TENG reached 138 V, 115 nC and 2.03 μA, respectively. This study provides an attractive power supply strategy for machine-mounted sensors of the rotating machinery, and the rotational speed measurement test also suggests the potential application of the R-TENG as a self-powered rotational speed sensor.

## 1. Introduction

Rotating shaft systems are widely used in industrial equipment, such as in marine main engines and in wind-power generation. The shaft power of a marine propeller-shafting system is the most important performance parameter of marine power plants, which can be measured by the torque of the shafting system. Torsional oscillation of the shafting can cause fatigue fractures, and excessive torque can cause deformation of the rotating shaft, posing a safety hazard [1]. Therefore, the monitoring of various parameters of the rotating shaft system can improve the reliability of the equipment and can effectively avoid accidents caused by failure. The monitoring of shafting mainly focuses on the measurement of parameters, including vibration, torque and temperature [2,3]. The measurement methods can be divided into the noncontact type and contact type. Noncontact measurement can be used to monitor the axial displacement, fatigue crack and rotational speed [4]. As for torque measurement and detection of the internal temperature of the shaft system, the contact measurement, the installation of corresponding sensors on the shaft is required. Some parameters such as the torsional vibration of the rotating shaft can be measured by means of either contact or noncontact measurements.

In general, the contact sensors mounted on the shaft system are usually powered by battery, and the battery rotates along with the rotating shaft, with the data being transmitted by wireless transmission. Due to the limited power-supply capacity of the battery, the battery needs to be replaced regularly, which does not meet the long-term monitoring requirement. The equipment shutdown caused by battery replacement interrupts the production plan and damages economic interests [5,6]. Therefore, the monitoring parameters of the rotating shaft currently tend to adopt noncontact measurement, and the monitoring parameters that require contact measurement are often ignored due to the power supply difficulty. In order to solve the power-supply problem of machine-mounted sensors, an inductively coupled power transfer (ICPT) technology device on the basis of the electromagnetic induction principle is proposed, which can free the sensor from dependence on the battery [7,8]. However, the inductive power supply device needs to rely on an external power supply to create an alternating magnetic field for electricity generation. Narrow space in industrial sites makes the installation difficult, and the cost of the ICPT system is relatively high, which is not suitable for large-scale applications. With the demand for sensor nodes of rotating machinery growing dramatically in the era of the Internet of Things (IoT), the conventional power supply method using a battery and inductive system cannot meet the extensive monitoring requirements. Miniaturized, low-cost and low-energy-consumption power supply methods must be proposed. 

The triboelectric nanogenerator has emerged as a promising energy harvesting technology since it was first proposed in 2012 by Wang et al. [9]. Conventional mechanical energy harvesting relies on technologies such as piezoelectrics [10,11,12], electrostatics [13] and electromagnetics [14]. Owing to its low cost, simple manufacturing process and widespread solutions for materials, the invention of the triboelectric nanogenerator provides an attractive approach for converting mechanical energy into electricity [15,16,17,18,19,20,21]. In recent years, lots of research has been conducted on adopting TENG for harvesting rotational mechanical energy. Lin et al. [22] reported on a segmentally structured disk triboelectric nanogenerator, which is an efficient way to harvest rotational mechanical energy, but the material wear of this method is severe. Yang et al. [23] reported a fully packaged, rolling hybrid triboelectric and electromagnetic nanogenerator to harvest rotational energy. Jiang et al. [24] proposed a water-wheel-type triboelectric nanogenerator for power generation using a slowly flowing river. Kim et al. [25] and Han et al. [26] successfully demonstrated a triboelectric nanogenerator for efficiently harvesting rotational mechanical energy by means of wind drive. Although extensive efforts have been made to harvest rotational mechanical energy into electricity by triboelectric nanogenerator technology, its structural design and suitable application scenarios still require further research.

In this study, we propose a freestanding mode ring-type triboelectric nanogenerator (R-TENG) to collect rotational mechanical energy and measure the rotational speed. The R-TENG is composed of a ring-type container unit, an end cover and polytetrafluoroethylene (PTFE) cylinders. The PTFE cylinders are placed in the ring-type container unit, and a pair of aluminum strips are coated on the inner and outer walls as one electrode. The working part of the designed R-TENG is encapsulated and cannot be affected by external humidity or by other environmental factors. When the shaft rotates, the combination of gravity and friction drives the PTFE cylinders to roll, and relative movement exists between the electrode and the PTFE cylinders, thereby producing output electricity periodically. The motion law of the PTFE cylinders is also investigated to elucidate the working mechanism of the R-TENG. Most high-efficiency rotating triboelectric nanogenerators require a fixed end to create relative motion between triboelectric materials [27,28,29], which requires extra work for installation. Compared to conventional rotating TENGs, the proposed R-TENG with a compact structure can be installed on the rotating shaft directly without extensive modification to the rotating machinery. Rotational speed measurement by the voltage signal processing of the R-TENG was also conducted, and the error rate of the R-TENG detection speed was within 1.5%, showing its potential ability to measure rotational rate. Using a reasonable energy-harvesting circuit design, the R-TENG can supply power to temperature and torque sensors, realizing the self-powered and self-sensing capability of the electronic device. This study provides an efficient power supply strategy for the contact measurement of rotating machinery.

## 2. Experimental Section

### 2.1. Fabrication of the R-TENG

In this experiment, the PTFE cylinder used was produced by 3M Company (Saint Paul, MN, USA) and was cut into four different sizes by machine tool cutting. The ring-type container unit and the corresponding end cover were designed by CATIA 3D drawing software and were made of composite resin using stereolithography (SLA) 3D printing technology. The end cover and the container unit were assembled with screws. The diameters and heights of the four different sizes of the PTFE cylinders were 18 mm × 20 mm, 15 mm × 20 mm, 12 mm × 20 mm and 9 mm × 20 mm. For the R-TENG with PTFE cylinders of different diameters, the inner radius of the container unit should remain unchanged, and the outer radius of the container unit and of the working space increases with the cylinder’s diameter increases. To ensure that the PTFE cylinders roll normally in the working space, there should be an air gap (approximately 3 mm) between the wall of the working space and the PTFE cylinder. Taking an R-TENG with 18 mm-diameter cylinders as an example, the outer radius, inner radius and height of the container unit are 84.5 mm, 50 mm and 24 mm, and the outer radius, inner radius and height of the working space in the container unit are 74.5 mm, 53 mm and 22 mm, respectively. Two aluminum strips (50 μm in thickness) with dimensions of 158 mm × 22 mm and 222 mm × 22 mm were attached with identical radian onto the inner and outer walls of the working space, respectively, to form one electrode, and they were connected by a wire. Finally, the R-TENG consists of two symmetrical electrodes.

### 2.2. Electrical Measurement of the R-TENG

In the experiment, a speed-adjustable DC motor with a maximum speed of 500 rpm was selected. A standard series of integrated conductive slip rings (MOFLON, Shenzhen, China) with an outer diameter of 185 mm and a through-hole diameter of 100 mm was selected to transmit the electrical output of the rotating R-TENG to the probe of the electrometer. The output values of the open-circuit voltage, transferred charge and short circuit current were measured using a programmable electrometer (Keithley Model 6514) through a data acquisition system (NI PCI-6220, Texas, USA) and a software program developed by LabVIEW (Texas National Instruments, Austin, TX, USA) that visualizes the output of the signal. 

## 3. Results and Discussion

### 3.1. Structure and Working Mechanism of the R-TENG

**Figure 1:**
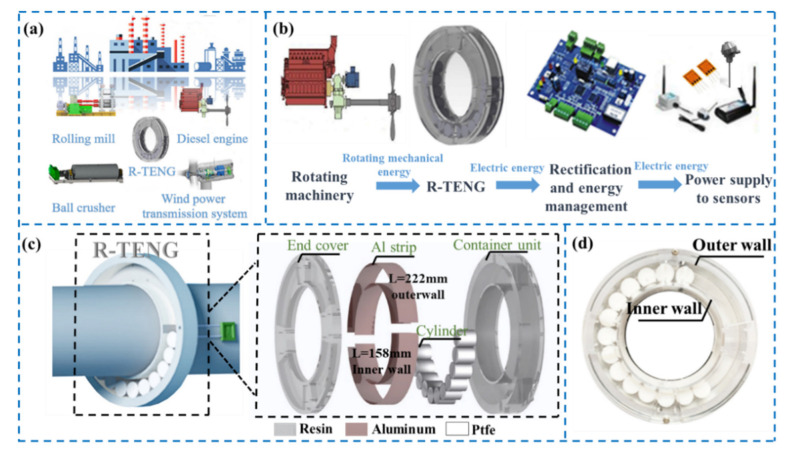
The structural design of the R-TENG: (**a**) potential application scenarios of the R-TENG in rotational mechanical energy harvesting; (**b**) schematic illustration of the rotational mechanical energy harvesting and power management system; (**c**) schematic illustration of the R-TENG mounted on a rotating shaft as a self-powered thermometer and the components of the designed R-TENG; (**d**) photograph of an assembled R-TENG.

Figure 1 depicts the structural design of the R-TENG, which can be used in various rotating machinery systems such as diesel engines and rolling mills for rotational mechanical energy harvesting, as presented in Figure 1a. Figure 1b displays the schematic illustration of the rotational mechanical energy harvesting and power management system. The designed ring-type TENG converts rotational mechanical energy to electricity, and the electricity is rectified by the energy management module, ultimately supplying power to machine-mounted sensors. Figure 1c presents the schematic illustration of the R-TENG mounted on the rotating shaft as a self-powered thermometer, and an enlarged view in the inset exhibits the structural design of the R-TENG. The R-TENG is composed of a container unit, an end cover and PTFE cylinders. The PTFE cylinders are placed in the working space of the container unit, and the container unit is assembled with the end cover by screws. A pair of aluminum strips (158 × 22 mm and 222 × 22 mm, 50 μm in thickness) is parallelly coated on the inner and outer walls of the working space and connected by a wire acting as one electrode. Two lead wires acting as electrodes connect to the two input ends of the slip ring through the holes of the container unit. Then, the container unit is mounted onto the rotating shaft by tightening the screws from the protruding parts. Figure 1d shows the photograph of an assembled R-TENG. 

**Figure 2:**
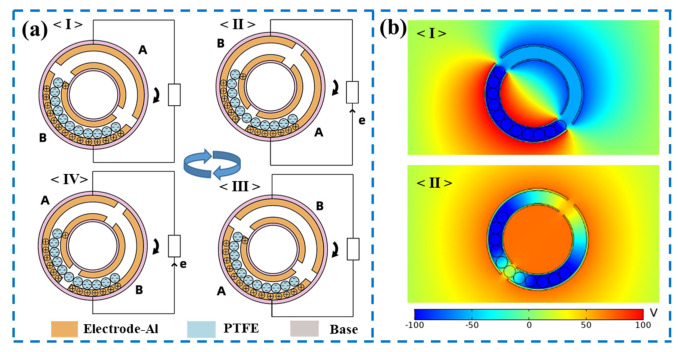
Working mechanism of the R-TENG: (**a**) schematic illustration of the working mechanism of the R-TENG; (**I**) cylinders fully overlapping electrode B; (**II**) cylinders getting in touch with electrode A; (**III**) cylinders fully overlapping electrode A; (**IV**) cylinders getting in touch with electrode B; (**b**) electrical potential distributions at different positions using COMSOL Multiphysics; (**I**) cylinders fully overlapping one electrode; (**II**) equivalent charge induced on two electrodes.

The R-TENG operates in the sliding freestanding mode using PTFE cylinders as a freestanding triboelectric layer. Once the shaft rotates, the PTFE cylinders will roll along the walls inside the working space of the container unit. After several revolutions of the R-TENG, the motion state of the PTFE cylinders will remain stable under the combination of gravity and friction. Moreover, it can be observed that the contact between the PTFE cylinders and the electrodes is mainly on the outer wall of the working space. Due to the higher electronegativity of PTFE than aluminum, the surface of the PTFE cylinder is negatively charged upon contact with the aluminum strip, while an equivalent amount of positive charges generate on the aluminum strip. Therefore, the working mechanism of the R-TENG for converting mechanical energy into electricity can be illustrated in a full rotation cycle in Figure 2a. In the initial state, all PTFE cylinders are in contact with electrode B, as plotted in Figure 2a(I). At this point, the difference in potential between the two electrodes reaches a maximum value according to the working principle of the freestanding triboelectric nanogenerator [30], and no current flows through the external circuit because of electrostatic equilibrium. As the R-TENG rotates clockwise with the shaft, PTFE cylinders gradually come into contact with electrode A, a difference in potential is formed between the two electrodes, and thus charges begin to flow through the external circuit, as plotted in Figure 2a(II). When PTFE cylinders completely overlap electrode A, the system attains electrostatic equilibrium again, and thus no current flows through the external circuit, as plotted in Figure 2a(III). As the R-TENG continues to rotate, the difference in potential between two electrodes is reversed, thus opposite transferred charge flowing through the external circuit, as plotted in Figure 2a(IV). These four states are periodically repeated as R-TENG continues to rotate, producing an alternating current through the external circuit.

To further demonstrate the proposed principle, the finite element method (via COMSOL Multiphysics) is utilized to simulate the electrical potential profiles in the R-TENG. The structure of the simulation model is the same size as that of the manufactured device. When the PTFE cylinders are in full contact with either of the aluminum strip electrodes, the difference in potential between the two electrodes reaches a maximum value of about 160 V, as illustrated in Figure 2b(I). When the amount of electrostatic charge generated by the contact between the two electrodes and the PTFE cylinders is equivalent, there exists no difference in potential between the two electrodes, as illustrated in Figure 2b(II).

To obtain a physical model for the electrical performance of the R-TENG, all PTFE cylinders are treated as a freestanding layer. According to the theory of the sliding-mode freestanding TENG, the V-Q-α relation of the R-TENG can be expressed as follows:

(1) $$V=R\frac{dQ}{dt}=-\frac{Q}{C_{\left(\alpha \right)}}+V_{oc}\left(\alpha \right)$$

(2) α(t)=ωt

where R, *C*, *V_oc_*, *α* and *ω* are the resistance, capacitance, open-circuit voltage, rotation angle and angular velocity of the R-TENG. Under the short-circuit condition, total triboelectric charges on aluminum film 1 and aluminum 2 in a small region of dα are expressed in Equations (3) and (4), and then *Q_sc,final_* and *I_sc_* are deduced, as presented in Equations (5) and (6).

(3) $${\mathrm{dQ}}_{1}=\frac{\sigma hd\alpha }{1+\frac{C_{2}\left(\alpha \right)}{C_{1}\left(\alpha \right)}}$$

(4) $${\mathrm{dQ}}_{2}=\frac{\sigma hd\alpha }{1+\frac{C_{1}\left(\alpha \right)}{C_{2}\left(\alpha \right)}}$$

(5) $$Q_{sc,final}=\int_{0}^{\theta_{l}}\frac{\sigma hd\alpha }{1+{\left(\frac{C_{2}\left(\alpha \right)}{C_{1}\left(\alpha \right)}\right)}_{\alpha =\theta_{l}+\theta_{g}}}-\int_{0}^{\theta_{l}}\frac{\sigma hd\alpha }{1+{\left(\frac{C_{2}\left(\alpha \right)}{C_{1}\left(\alpha \right)}\right)}_{\alpha =0}}$$

(6) $$I_{sc}=\frac{Q_{sc}}{\Delta t}=\frac{Q_{sc}}{\frac{d\alpha }{\omega }}=\frac{Q_{sc}\times \omega }{d\alpha }$$

where we set the charge density as *σ*, the angle of one electrode as *θ_l_*, the height of the cylinder as *h*, the gap angle between two electrodes as *θ_g_* and ∆*t* as the time used of transferred charge.

### 3.2. Output Performance of the R-TENG

**Figure 3:**
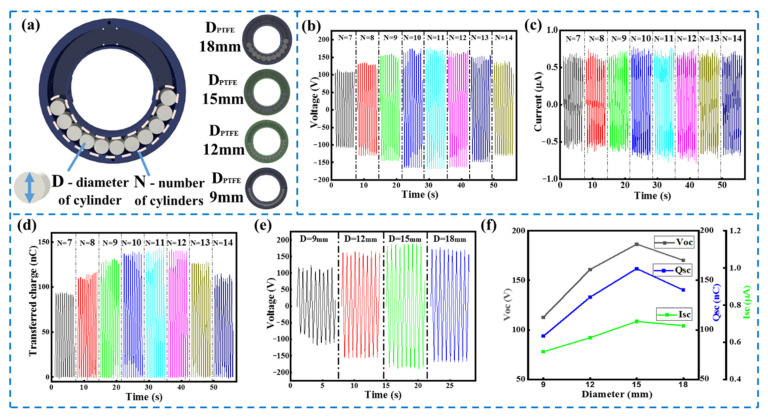
Electrical output of the R-TENG: (**a**) schematic illustration of structural parameters of the R-TENG; (**b**) output voltage; (**c**) current and (**d**) transferred charge of the R-TENG with the increasing number of cylinders generated at 100 rpm; (**e**) output voltage of R-TENG with PTFE cylinders of different diameters; (**f**) trend chart of the electrical performance of the R-TENG with cylinders of different diameters at 100 rpm.

In this study, to achieve an optimal output performance of the R-TENG, the structural parameters of the device were systematically investigated. Figure 3a depicts the schematic illustration of the structural parameters of the R-TENG. Firstly, the influence of the number of PTFE cylinders on the electrical output of the R-TENG was measured with a different number of cylinders at 100 rpm. It can be clearly observed from Figure 3b,d that, with the increasing number of cylinders, both the output peak voltage and the transferred charge of the R-TENG increase first and then decrease, and the device achieves optimal electrical performance when the number of PTFE cylinders equals 11. For the R-TENG used in this experiment (D_PTFE_ = 18 mm), 11 cylinders can completely overlap one aluminum electrode. This output result is consistent with the working principle of the freestanding mode triboelectric nanogenerator that the difference in potential and the transferred charge reach their maximum values as either of the electrodes is in full contact with the PTFE cylinders. When the number of cylinders is greater than 11, one electrode is completely covered by 11 PTFE cylinders, and there exists contact between the other electrode and extra PTFE cylinders, leading to a decline in the difference in potential between the two electrodes. When the number of cylinders is less than 11, the PTFE cylinders cannot cover the entire single electrode, and the corresponding contact area is decreased, leading to a decline in the difference in potential between the two electrodes as well. The variation of the peak current with the number of cylinders is not obvious, as shown in Figure 3c. The reason for this is that the dielectric layer length, that is, the number of cylinders, mainly affects the duration of the transferred charge rather than the rate of change in the metal sliding freestanding triboelectric nanogenerator [30]. It can be observed from Figure S1 that the duration of the R-TENG’s peak current tends to increase first and then decrease approximately, which is consistent with the variation in the amount of transferred charge with the number of cylinders.

The influence of the PTFE cylinder’s diameter, another important structural parameter, on the electrical performance was also investigated. In the experiment, four different cylinder diameters with the same height (20 mm) were applied. In order to ensure the normal motion of the cylinder, there needs to be an air gap between the cylinder and the wall of the working space. The overall dimensions of R-TENG will increase with the increasing diameter of the cylinder, and the photos of the R-TENGs with different-diameter cylinders are shown in Figure S2. For the R-TENGs with different-diameter cylinders, the number of PTFE cylinders that can completely cover one electrode is selected in the experiment to obtain the optimal electrical output. Figure 3e,f presents the electrical output of the R-TENG with PTFE cylinders of different diameters at 100 rpm. It is seen that the peak voltage of the R-TENG increases first and then decreases, and the optimal output performances can be achieved as the diameter of the PTFE cylinder is 15 mm. The reason for this is that the electrical performance of the R-TENG is related to the charge generated by triboelectrification, which depends on the contact area and the friction force. As the diameter of the cylinder decreases, the effective contact area between the PTFE cylinders and the electrodes increases, as shown in Figure S3, which is beneficial for the charge induction. However, the weight of the PTFE cylinders also decreases with the cylinders’ decreasing diameter, and the corresponding friction force decreases, which is unfavorable to the charge induction [31]. There is a trade-off between an increase in the contact area and a reduction in the weight of the cylinder. Additionally, the experimental results showed that small-diameter PTFE cylinders are more likely to cross the highest point of the ring at a low rotational speed. Therefore, it is necessary to select a PTFE cylinder of a suitable diameter by considering the dimension, operating speed and electrical performance of the R-TENG.

**Figure 4:**
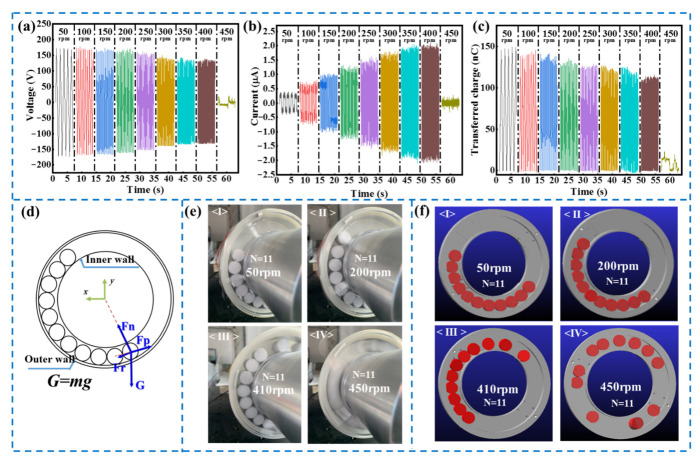
Rotational speed characteristics of the R-TENG: (**a**) output voltage, (**b**) current and (**c**) transferred charge of R-TENG with 11 PTFE cylinders at different rotational speeds; (**d**) schematic illustration of the dynamic analysis of the PTFE cylinders rolling in the container; (**e**) photograph of the rotating R-TENG at different rotational speeds; (**f**) kinetic simulation of PTFE cylinders at different rotational speeds via ADAMS.

To examine the relationship between the electrical output of the R-TENG and the rotational speed, a systematic measurement was performed at different rotational speeds of the R-TENG from 50 rpm to 450 rpm with 11 cylinders 18 mm in diameter. It is seen from Figure 4a,c that voltage and transferred charge decrease with increasing rotational speed, which may be attributed to the inefficient contact between the cylinders and electrodes at high rotational speeds. According to Equations (5) and (6), the transferred charge is not affected by speed theoretically. Despite a small decrease in transferred charge with increasing speed in the actual measurement, the degree of ω’s increase is much more significant than the degree of decline in *Q_sc_*, resulting in the current *I_sc_* steadily increasing, as shown in Figure 4b. Furthermore, it is noteworthy that the electrical output of the R-TENG decreases significantly at 450 rpm, which is due to the change in motion state of the PTFE cylinders that the relative movement between the PTFE cylinders and aluminum electrodes disappears at high rotational speeds.

The electrical output of the R-TENG is generated by triboelectrification following the relative contact between the PTFE cylinders and the electrodes. In the experiment, it is clearly observed that the motion state of PTFE cylinders changes with the rotational speed, thereby affecting the electrical performance of the R-TENG. Therefore, it is necessary to investigate the motion law of the cylinders with respect to rotational speed. Figure 4d presents the dynamic analysis of the PTFE cylinders rolling in the container unit as the device rotates. Photographs and videos of the PTFE cylinders in motion at different rotational speeds were taken with a digital camera, and the motion states of the cylinders were divided into three different states, as illustrated in Figure 4e and Video S1. In the first state (from 0 to 400 rpm), the position of the cylinders remains almost unchanged along the rotating direction with little disturbance, and the angular offset of the cylinders also increases with increasing rotational speed. In this state, the electrical output of the R-TENG follows the working principle mentioned in Section 3.1. In the second state (at about 410 rpm), there exist cylinders crossing the highest point of the R-TENG and dropping intermittently. In this state, the electrical output becomes unstable, and noise is generated by the impact. In the third state (at about 440 rpm), PTFE cylinders rotate along with the ring together, and no relative motion between the PTFE cylinders and the electrode exists, resulting in a significant decline in output performance. To support the experimental results, ADAMS was employed to study the motion law of the cylinders, and the kinetic simulations (at 50, 200, 410 and 450 rpm) are presented in Figure 4f. The simulation result that the motion of the cylinders goes through three states as well is consistent with the phenomenon observed in the experiment, illustrating that the triboelectric nanogenerator with a ring-type structure has a rotational speed limit in practical applications. Therefore, the operating speed of the R-TENG with 18 mm diameter cylinders is defined in the range of 0–400 rpm. Through experiments and simulation, it can be found that the friction force on the cylinder and the vibration at high speeds lead to the cylinder rotating along with the R-TENG together. Therefore, ensuring the shafting alignment and reducing the roughness of the cylinder and the electrodes can extend the operating speed range of the device. 

### 3.3. Demonstration

**Figure 5:**
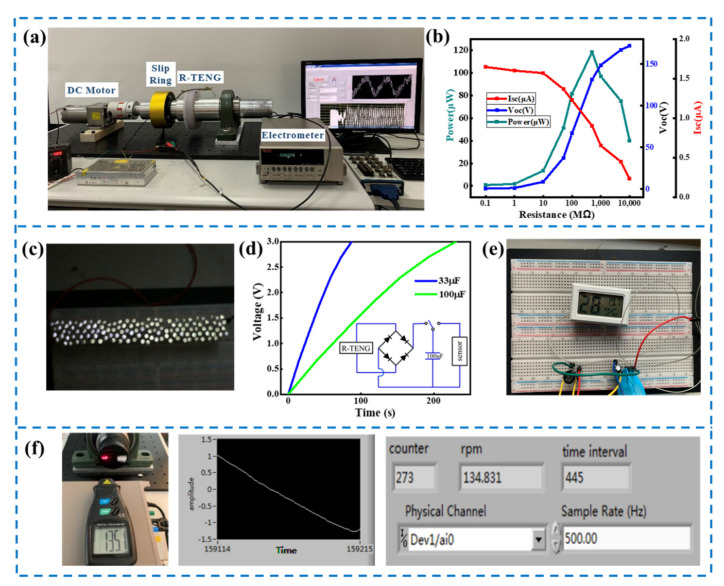
Demonstration of the R-TENG for energy harvesting and rotational speed measurement: (**a**) photograph of the test devices; (**b**) output voltage, current and power for the R-TENG with the resistance of the load; (**c**) photograph of 80 white LEDs powered by the R-TENG; (**d**) charging curve of different capacitors by the R-TENG electrical output; (**e**) photograph of a 100 µF capacitor charged and supplying power to thermometer; (**f**) tests of rotational speed measurement by output voltage signal of the R-TENG via LabVIEW.

Figure 5a presents the experimental setup, and an R-TENG with 18 mm diameter cylinders was applied in the demonstration. As illustrated in Figure 5b, the current, the voltage and the power of the R-TENG at 250 rpm were measured with various external load resistances, and a maximum output power of 107 µW was reached when the external resistance equaled the internal impedance. A total of 80 commercial light-emitting diodes (LEDs) was utilized as external loads of the R-TENG to verify its ability to drive the electronics, as illustrated in Figure 5c and Video S2. Furthermore, the R-TENG was also employed in conjunction with energy storage devices to power commercial electronic sensors. At a rotational speed of 400 rpm, the 33 µF and 100 µF capacitors can be charged by the R-TENG through a bridge rectifier from 0 to 3 V within 90 s and 230 s, respectively, as illustrated in Figure 5d. Then, the commercial thermometer was powered by the capacitor, as illustrated in Figure 5e. Since the output frequency is synchronized with the rotational speed, a LabVIEW program was developed to measure the rotational speed by calculating the time difference of the up-crossing zero points of the voltage signal, as illustrated in Figure S4. The rotational speed measurements via LabVIEW were carried out at different speeds, and the test results suggest that the rotational rate measured utilizing the R-TENG is consistent with the value measured with a commercial tachometer. Here, the speed deviation δ is defined as

(7) $$\delta =\left|\frac{x_{i}-X_{0}}{X_{0}}\right|$$

where *x_i_* is the speed measurement via LabVIEW, and the *X*_0_ is the speed measurement via the commercial speed sensor. The speed deviation was within 1.5%, which was obtained by conducting speed deviation analysis at different operating speeds. The reason for the deviation is that PTFE cylinders swing as R-TENG rotates, as can be found in Video S1. No obvious decline in the voltage of the R-TENG was found after continuous operation of 15,000 cycles, demonstrating the reliability of the device, as illustrated in Figure S5. The above-mentioned tests suggested the ring-type TENG as an efficient method to harvest rotational mechanical energy, and this concept provides a new strategy to supply power for electronics mounted on the rotating shaft, such as a torque sensor and an inner-temperature monitoring sensor.

## 4. Conclusions

In summary, we proposed a ring-type freestanding triboelectric nanogenerator, being mounted on a rotating shaft, which could harvest rotational mechanical energy. Such a ring-type triboelectric nanogenerator consists of an end cover, a container unit and multiple PTFE cylinders. With a fully packaged structure, the R-TENG could be used in harsh environments. At a rotational speed of 400 rpm, the R-TENG delivered an output voltage of up to 138 V, an output transferred charge of 115 nC and an output current of 2.03 µA. Through a systematical examination of the design parameters, it was found that the R-TENG achieves optimal performance as PTFE cylinders completely overlap one electrode and there exists a certain diameter of the cylinders to maximize electrical output. Due to the increase in the amount of transferred charge per unit time, increasing the rotational speed is generally beneficial to energy harvesting. According to the experimental and simulation research on the motion law of the cylinder, it was found that the R-TENG has a maximum operating speed limit. The demonstration showed that the R-TENG could directly light up 80 LEDs and charge a 100 µF capacitor to 3 V within 4 min. Therefore, the design of the R-TENG provides a promising strategy to supply power to the machine-mounted sensors of the rotating machinery. Furthermore, tests were conducted to measure the rotational speed by the voltage signal, suggesting its possible application as a self-powered tachometer.

## Data Availability

The data presented in this study are available on request from the corresponding author.

## Supplementary Materials

The following supporting information can be downloaded at: https://www.mdpi.com/article/10.3390/mi13040556/s1, Figure S1: The duration of the R-TENG’s peak current with an increasing number of PTFE cylinders; Figure S2: Photograph of the R-TENGs with PTFE cylinders of different diameters; Figure S3: Schematic illustration of change in contact area with cylinders of different diameters; Figure S4: Schematic diagram of the synchronization in voltage frequency and rota-tional speed; Figure S5: Continuous output performance of the R-TENG during 15,000 cycles; Video S1: Motion state of 11 PTFE cylinders at different rotational speeds; Video S2: Demonstration of the R-TENG to power 80 LEDs.

## References

[1] Liu C., Jiang D. (2020). Torsional vibration characteristics and experimental study of cracked rotor system with torsional oscillation. Eng. Fail. Anal..

[2] de Azevedo H.D.M., Araújo A.M., Bouchonneau N. (2016). A review of wind turbine bearing condition monitoring: State of the art and challenges. Renew. Sustain. Energy Rev..

[3] Presas A., Luo Y., Wang Z., Guo B. (2019). Fatigue life estimation of Francis turbines based on experimental strain measurements: Review of the actual data and future trends. Renew. Sustain. Energy Rev..

[4] Jeon I., Lim H.J., Liu P., Park B., Heinze A., Sohn H. (2019). Fatigue crack detection in rotating steel shafts using noncontact ultrasonic modulation measurements. Eng. Struct..

[5] Mongia C., Goyal D., Sehgal S. (2021). Vibration response-based condition monitoring and fault diagnosis of rotary machinery. Mater. Today Proc..

[6] Chethan C.R., Tewari V.K., Nare B., Kumar S.P. (2018). Transducers for Measurement of Draft and Torque of Tractor-implement System—A Review. Agric. Mech. Asia Afr. Lat. Am..

[7] Lee J.-Y., Chen H.-M., Huang L.-Y. (2015). Design of an Improved Type Rotary Inductive Coupling Structure for Rotatable Contactless Power Transfer System. MATEC Web Conf..

[8] Wang X., Wang A., Wang X. (2016). Modeling Analysis of Contactless Power Transfer System for Rotary Ultrasonic Machining. Appl. Mech. Mater..

[9] Fan F.-R., Tian Z.-Q., Wang Z.L. (2012). Flexible triboelectric generator. Nano Energy.

[10] Wang X. (2012). Piezoelectric nanogenerators—Harvesting ambient mechanical energy at the nanometer scale. Nano Energy.

[11] He P., Chen W., Li J., Zhang H., Li Y., Wang E. (2020). Keggin and Dawson polyoxometalates as electrodes for flexible and transparent piezoelectric nanogenerators to efficiently utilize mechanical energy in the environment. Sci. Bull..

[12] Wei X., Liu X., Zheng C., Zhao H., Zhong Y., Amarasinghe Y.W.R., Wang P. (2021). A piezoelectric power generator based on axisymmetrically distributed PVDF array for two-dimension vibration energy harvesting and direction sensing. Sustain. Energy Technol. Assess..

[13] Basset P., Galayko D., Paracha A.M., Marty F., Dudka A., Bourouina T. (2009). A batch-fabricated and electret-free silicon electrostatic vibration energy harvester. J. Micromech. Microeng..

[14] Glynne-Jones P., Tudor M.J., Beeby S.P., White N.M. (2004). An electromagnetic, vibration-powered generator for intelligent sensor systems. Sens. Actuators A Phys..

[15] Zhu G., Peng B., Chen J., Jing Q., Wang Z.L. (2015). Triboelectric nanogenerators as a new energy technology: From fundamentals, devices, to applications. Nano Energy.

[16] Xiao X., Zhang X., Wang S., Ouyang H., Chen P., Song L., Yuan H., Ji Y., Wang P., Li Z. (2019). Honeycomb Structure Inspired Triboelectric Nanogenerator for Highly Effective Vibration Energy Harvesting and Self-Powered Engine Condition Monitoring. Adv. Energy Mater..

[17] Li R., Zhang H., Wang L., Liu G. (2021). A Contact-Mode Triboelectric Nanogenerator for Energy Harvesting from Marine Pipe Vibrations. Sensors.

[18] Wu C., Huang H., Li R., Fan C. (2020). Research on the Potential of Spherical Triboelectric Nanogenerator for Collecting Vibration Energy and Measuring Vibration. Sensors.

[19] Zhao T., Xu M., Xiao X., Ma Y., Li Z., Wang Z.L. (2021). Recent progress in blue energy harvesting for powering distributed sensors in ocean. Nano Energy.

[20] Du T., Zuo X., Dong F., Li S., Mtui A.E., Zou Y., Zhang P., Zhao J., Zhang Y., Sun P. (2021). A Self-Powered and Highly Accurate Vibration Sensor Based on Bouncing-Ball Triboelectric Nanogenerator for Intelligent Ship Machinery Monitoring. Micromachines.

[21] Wang Y., Liu X., Wang Y., Wang H., Wang H., Zhang S.L., Zhao T., Xu M., Wang Z.L. (2021). Flexible Seaweed-Like Triboelectric Nanogenerator as a Wave Energy Harvester Powering Marine Internet of Things. ACS Nano.

[22] Lin L., Wang S., Xie Y., Jing Q., Niu S., Hu Y., Wang Z.L. (2013). Segmentally Structured Disk Triboelectric Nanogenerator for Harvesting Rotational Mechanical Energy. Nano Lett..

[23] Yang H., Wang M., Deng M., Guo H., Zhang W., Yang H., Xi Y., Li X., Hu C., Wang Z. (2019). A full-packaged rolling triboelectric-electromagnetic hybrid nanogenerator for energy harvesting and building up self-powered wireless systems. Nano Energy.

[24] Jiang D., Guo F., Xu M., Cai J., Cong S., Jia M., Chen G., Song Y. (2019). Conformal fluorine coated carbon paper for an energy harvesting water wheel. Nano Energy.

[25] Kim D., Tcho I.-W., Choi Y.-K. (2018). Triboelectric nanogenerator based on rolling motion of beads for harvesting wind energy as active wind speed sensor. Nano Energy.

[26] Han Q., Ding Z., Sun W., Xu X., Chu F. (2020). Hybrid triboelectric-electromagnetic generator for self-powered wind speed and direction detection. Sustain. Energy Technol. Assess..

[27] Qian J., Jing X. (2018). Wind-driven hybridized triboelectric-electromagnetic nanogenerator and solar cell as a sustainable power unit for self-powered natural disaster monitoring sensor networks. Nano Energy.

[28] Bi M., Wu Z., Wang S., Cao Z., Cheng Y., Ma X., Ye X. (2020). Optimization of structural parameters for rotary freestanding-electret generators and wind energy harvesting. Nano Energy.

[29] Wu Z., Wang S., Cao Z., Ding R., Ye X. (2021). Rotary disk multi-phase freestanding-electret generator with enhanced power and low ripple output. Nano Energy.

[30] Niu S., Liu Y., Chen X., Wang S., Zhou Y.S., Lin L., Xie Y., Wang Z.L. (2015). Theory of freestanding triboelectric-layer-based nanogenerators. Nano Energy.

[31] Pan S., Yin N., Zhang Z. (2018). Time- & Load-Dependence of Triboelectric Effect. Sci. Rep..

