# Supplementary material for: A Ring-Type Triboelectric Nanogenerator for Rotational Mechanical Energy Harvesting and Self-Powered Rotational Speed Sensing
Source: Micromachines (Basel). 2022 Mar 31;13(4):556. doi: 10.3390/mi13040556 (PMC9025401; doi:10.3390/mi13040556)

**Supporting information**

**A ring type triboelectric nanogenerator for rotational mechanical energy harvesting and self-powered rotational speed sensing**

Supporting Videos

**Video S1**. Motion state of 11 PTFE cylinders at different rotational speeds

**Video S2**. Demonstration of the R-TENG to power 80 LEDs

Supporting Figures

**Figure S1.** The duration of the R-TENG’s peak current with increasing number of PTFE cylinders.

**Figure S2.** Photograph of the R-TENGs with PTFE cylinders of different diameters.


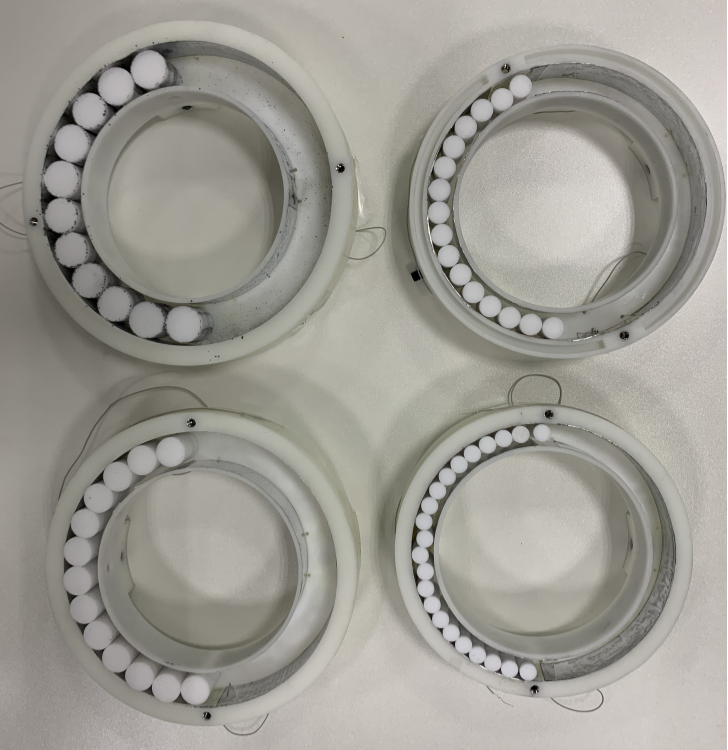


**Figure S3.** Schematic illustration of change in contact area with cylinders of different diameters.


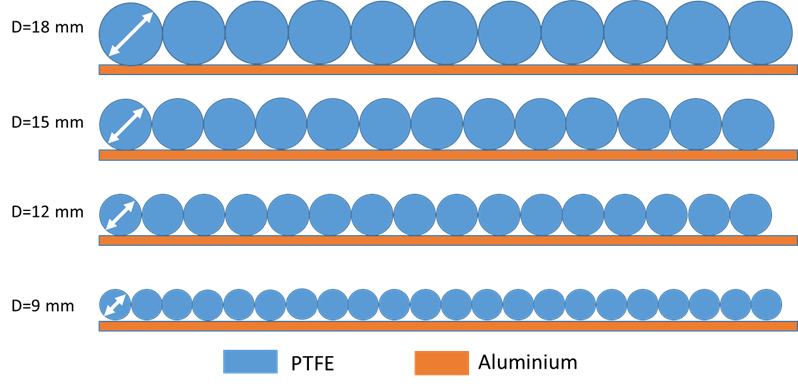


**Figure S4.** Schematic diagram of the synchronization in voltage frequency and rotational speed.

**Figure S5.** Continuous output performance of the R-TENG during 15000 cycles.


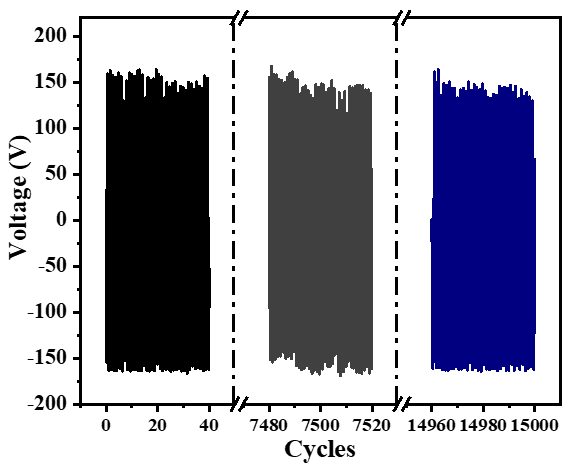

Supplement: Supplementary file 1 [file micromachines-13-00556-s001.zip › micromachines-1646449 supplementary/micromachines-1646449-Supporting Information.docx]
